# Enhanced photocatalytic degradation of tetracycline hydrochloride over Au-doped BiOBr nanosheets under visible light irradiation

**DOI:** 10.1371/journal.pone.0273169

**Published:** 2022-08-26

**Authors:** Chu-Ya Wang, Xin Fang, Qi Zeng, Heng-Deng Zhou, Yongze Lu

**Affiliations:** School of Energy and Environment, Southeast University, Nanjing, China; Shoolini University, INDIA

## Abstract

Bismuth(III) oxybromide (BiOBr) is a typical photocatalyst with a unique layered structure. However, the response of BiOBr to visible light is not strong enough for practical application. Moreover, the charge separation efficiency of BiOBr still needs to be improved. In this study, series of Au-doped BiOBr photocatalysts was prepared through a facile one-step hydrothermal method. The as-prepared Au_0.3_-BiOBr nanosheets exhibited an excellent electrochemical performance. The charge separation efficiency of Au_0.3_-BiOBr nanosheets was enhanced by 18.5 times compared with that of BiOBr. The intrinsic photocatalytic activity of Au_0.3_-BiOBr nanosheets in the degradation of tetracycline hydrochloride was approximately twice higher than that of BiOBr under visible light irradiation. In addition, three pathways were identified for the photocatalytic degradation and mineralization of tetracycline hydrochloride, which involve four reactions: hydroxylation, demethylation, ring opening and mineralization. Accordingly, this study proposes a feasible and effective Au-doped BiOBr photocatalyst, and describes a promising strategy for the design and synthesis of high-performance photocatalysts.

## Introduction

Due to the rapid population and economic growth worldwide, energy shortages and environmental pollution have become increasingly serious. Photocatalytic technology has a great potential application in environmental protection and energy development. Photocatalytic degradation of organic pollutants is an advanced oxidation technology, in which clean and sustainable solar energy could be used as the driving force to completely mineralize organic pollutants into CO_2_ and H_2_O [1–5]. Among various semiconductor photocatalysts, bismuth(III) oxybromide (BiOBr) is a promising photocatalyst with a unique layered structure, composed of stacked [Bi_2_O_2_]^2+^ layers and interlaced halogen ions [Br_2_]^2-^ [6–9]. The layered structure has a large internal space, which can effectively promote the polarization of related atoms and orbitals. In addition, the electronic field between [Bi_2_O_2_]^2+^ and [Br_2_]^2-^ layers can promote the separation and transfer of photogenerated electron-hole pairs, and thus improve the photocatalytic effect [10–12]. Although BiOBr has a suitable band gap of 2.7 eV, its response to visible light is not strong enough for practical applications [8]. Moreover, there is also a need to further improve the charge separation efficiency of BiOBr.

In recent decades, many research efforts have been focused on how to improve the light absorption of BiOBr. An effective strategy is to enhance the visible light photocatalytic activity by developing a BiOBr-based heterojunction, such as ZnO/BiOBr [13], TiO_2_/BiOBr [14], BiOBr/BiMoO_6_ [15] and Ag_2_CO_3_/BiOBr/CdS [16]. However, it is difficult to achieve a highly homogeneous distribution of constituents when constructing a heterojunction, which makes the preparation of a catalyst more complicated. In addition, introducing oxygen vacancies as lattice defects and varying the morphology have been undertaken to improve the photocatalytic performance of BiOBr [17–20]. However, many shortcomings remain to be overcome. For example, tailoring the morphology does not change the intrinsic light absorption property of BiOBr, and oxygen vacancies might result in a long reaction times [21]. In addition, the enhancement of the charge separation efficiency of BiOBr is also important for its photocatalytic activity.

Doping modification is a promising strategy to tailor the band structure of a semiconductor without substantially changing the host crystal structure. Previous studies reported the successful synthesis of Ag-doped BiOBr with flower-like microsphere structure by a simple solvothermal method [22]. The doping modification of BiOBr with Ag improved light absorption and charge separation efficiency of the catalyst, which resulted in a higher photocatalytic activity. Moreover, the doping modification of BiOBr with self-assembled hollow-microsphere structure with Fe could also improve the photocatalytic ability [23]. Based on these studies, and considering the excellent electrochemical performance of Au, it is theoretically possible that doping modification with Au might improve the charge separation efficiency and photocatalytic performance of BiOBr.

With the rapid development of photocatalysts, an important problem has been studied, that is, the separation of catalysts from the treated system after reaction, and this problem greatly restricts their wide application. It has been reported in the past that introducing magnetic materials into the photocatalyst will facilitate recovery using an external magnetic field [24, 25]. Therefore, the non-magnetic Au-doped BiOBr photocatalyst in this work needs to be combined with magnetic materials in future studies to improve the application of BiOBr-based photocatalytic nanomaterials.

In this study, a series of Au-doped BiOBr nanosheets were synthesized through a simple hydrothermal route. The morphology, crystal structure and energy band structure of Au-doped BiOBr were systematically characterized by various physicochemical techniques. Then, the photoelectric characteristics of Au-doped BiOBr were systematically analyzed. In addition, the photocatalytic performance without dye-sensitization was investigated using tetracycline hydrochloride (TH), a typical non-dye antibiotic pollutant, as the target pollutant. Also, the photocatalytic activity of Au-doped BiOBr under visible light irradiation was investigated. Additionally, the main active species in the degradation process were identified. Furthermore, the intermediates of TH degradation were also identified, and the degradation pathway of TH was elucidated. Based on these results, an effective strategy for the design and synthesis of high-performance visible photosensitive catalysts is proposed.

## Materials and methods

### Materials

Ethylene glycol and gold(III) chloride trihydrate (HAuCl_4_⋅3H_2_O) were purchased from Sinopharm Chemical Reagent Co., Ltd. (Shanghai, China) and Shanghai Macklin Biochemical Co., Ltd. (Shanghai, China), respectively, Bismuth nitrate pentahydrate (Bi(NO_3_)_3_·5H_2_O), ammonium bromide (NH_4_Br), distilled water, and other reagents were obtained from Aladdin Reagent Co., Ltd. (Shanghai, China). The purchased chlorauric acid solids (HAuCl_4_⋅3H_2_O) were formulated into a reserve solution at a concentration of 30 g/ L. All chemicals used in this study were of analytical-grade and were used directly without any further purification.

### Synthesis of Au-doped BiOBr photocatalyst

The Au-doped BiOBr nanomaterial was synthesized by a typical hydrothermal procedure, as follows. First, 0.970 g (2 mmol) of Bi(NO_3_)_3_·5H_2_O was added into 5 mL ethylene glycol, and mixed by continuous ultrasonication until a homogeneous solution was obtained. Additionally, 0.196 g (2 mmol) of NH_4_Br was dissolved into 30 mL of distilled water, and the mixture was continuously stirred for 5 min to obtain a clear suspension. Then, different amounts of HAuCl_4_ reserve solution (2.65×10^−2^, 5.3×10^−2^ and 7.95×10^−2^ mmol) were added into the above-mentioned suspension, and the obtained solutions were designated as Au_0.3_-BiOBr, Au_0.6_-BiOBr and Au_0.9_-BiOBr, respectively. Afterwards, the mixture was transferred into a 50-mL Teflon-lined reactor, and heated at 160°C for 12 h. Subsequently, after cooling to room temperature naturally, the product powder was collected by centrifugation and each samples was washed three times with distilled water and anhydrous ethanol to remove the residuals and impurities. Eventually, the samples were dried in vacuum at 80°C for 10 h. For pure BiOBr, the synthetic procedure was the same as that for Au-doped BiOBr, but without adding HAuCl_4_ solution.

### Characterization

The crystallinity of the samples was determined by X-ray diffraction (XRD) using a Bruker D8 Advance diffractometer (Bruker AXS GmbH, Karlsruhe, German) equipped with a monochromatized Cu Kα radiation (λ = 1.541874 Å) source. The morphology of the samples was mexamined by (SEM), using a JSM-700F scanning electron microscope (JEOL Ltd., Tokyo, Japan) and the elements were confirmed by energy-dispersive X-ray spectroscopy (EDS). The microscopic morphology of the product was characterized by transmission electron microscopy (TEM), using a JEM 2100F transmission electron microscope (JEOL Ltd.). X-ray photoelectron spectroscopy (XPS) was performed on a Thermo Scientific K-Alpha X-ray Photoelectron Spectrometer system (Thermo Fisher Scientific Inc., Waltham, MA, USA) to determine the chemical composition and the valence potential of the as-prepared samples. The optical band gap of the samples was measured by diffuse reflectance spectroscopy (DRS) using a Shimadzu UV-3600i Plus ultraviolet/visible/near infrared (UV/Vis/NIR) spectrometer (Shimadzu Corporation, Kyoto, Japan). The Au content in the sample was analyzed by inductively coupled plasma emission spectroscopy (ICP-MS), Agilent 7800 ICP-MS system (Agilent Technologies Inc., Santa Clara, CA, USA). The surface area was measured by gas absorption on a Micromeritics APSP 2460 surface area and porosity analyzer (Micromeritics Corporation, Norcross, GA, USA), using the Brunauer-Emmett-Teller (BET) method. TH degradation products were identified by high performance liquid chromatography-mass spectrometry (HPLC-MS) using the Ultimate^TM^ 3000 HPLC—Q Exactive System (Thermo Fisher Scientific Inc.).

### Electrochemical measurements

All electrochemical characterizations were performed on a CHI760E electrochemical workstation (CH Instrument Co., Shanghai, China) using a three-electrode system. A Pt wire and an Ag/AgCl (KCl, 3 M) electrode were used as the counter electrode and the reference electrode, respectively. Quartz glass was used in the photocurrent experiment, and other parts were ordinary glass products. The working electrodes used in the Electrochemical impedance spectroscopy (EIS) measurements, Mott-Schottky plots and photocurrent responses tests were prepared as follows: 5 mg of catalyst was ultrasonically dispersed in 1 mL of methanol, and 10 μL of Nafion solution was added, mixed well and dripped onto both the glassy carbon electrode and F-doped SnO_2_ (FTO) glass. The EIS measurements were performed in 0.05 M K_3_[Fe(CN)_6_] and K_4_[Fe(CN)_6_] electrolyte solution at an alternating current frequency of 1~10^6^ Hz and a voltage amplitude of 5 mV. Mott-Schottky plots were performed in 0.1 M Na_2_SO_4_ electrolyte solution at a frequency of 1,000 Hz and an alternating current voltage amplitude of 5 mV. Photocurrent measurements were performed in 0.1 M Na_2_SO_4_ electrolyte solution with a bias voltage of 0.5 V.

### Photocatalytic activity evaluation

The photocatalytic degradation activity of the samples was measured using a CHF-XM500, 500 W Xenon light source (PerfectLight, Beijing, China) with a 420 nm cutoff filter at room temperature. The target pollutant was 50 mg/L of TH solution. In a typical degradation process for each experiment, 10 mg of photocatalyst powder was added into 50 mL of the TH solution, and the mixture was continuously stirred for 20 min in the dark to achieve adsorption-desorption equilibrium. At a specific time interval, 0.5 mL of the solution was taken out from the reaction system and immediately centrifuged. After that, the TH concentration of the obtained samples was measured by HPLC using a Hitachi Primaide HPLC system (Hitachi Ltd.). The temperature of the chromatographic column was 30°C. The mobile phase was composed of deionized water (containing 0.1% formic acid) and acetonitrile with a volume ratio of 2:3, and the flow rate was set at 0.5 mL/min. All experiments were performed in duplicate.

## Results and discussion

### Characterizations

**Fig 1:**
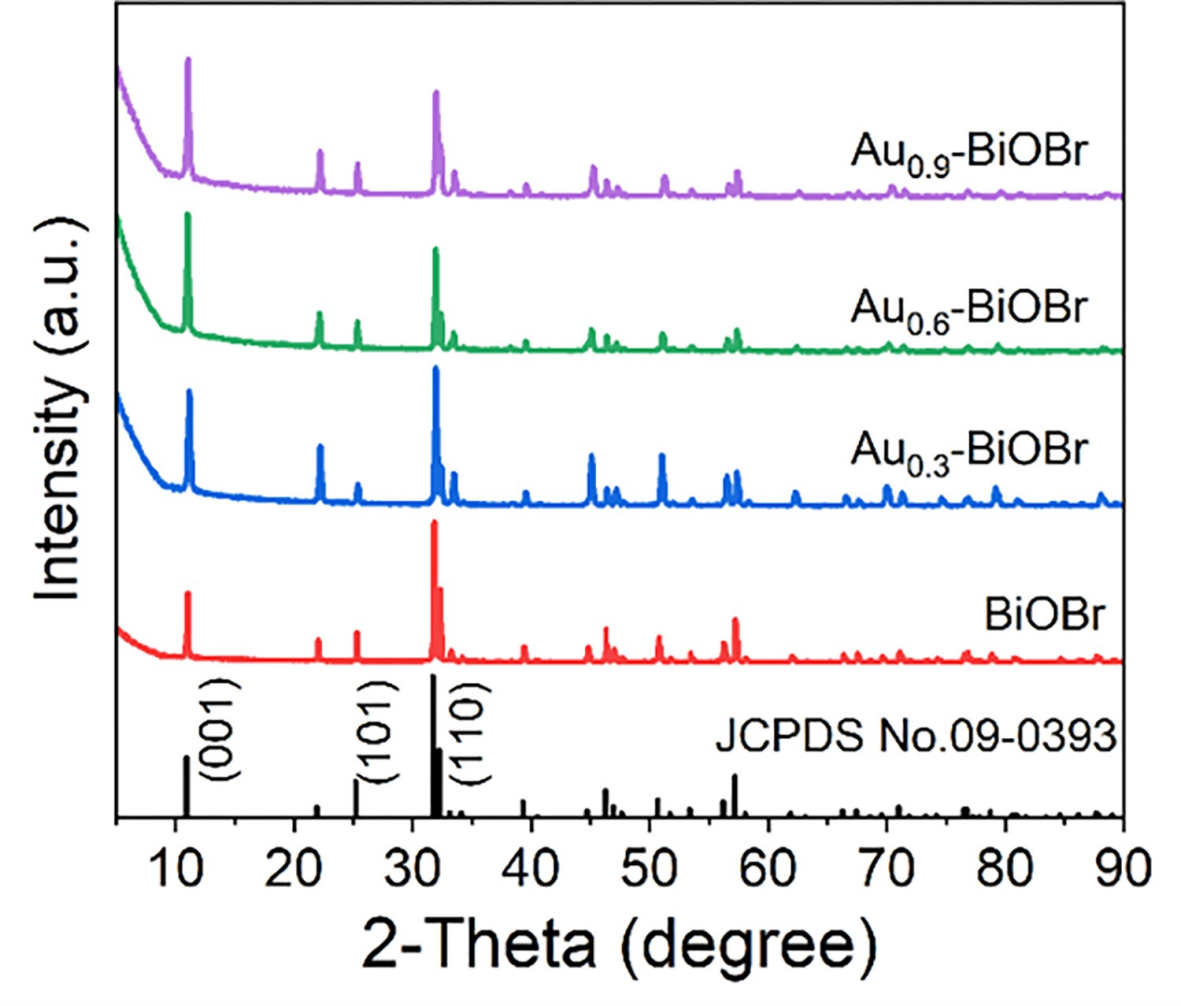
XRD patterns of BiOBr and Au-modified BiOBr, respectively.

The phase of the as-prepared products was determined by XRD analysis. All the diffraction peaks in the XRD spectra (Fig 1) could be indexed to BiOBr (JCPDS No. 09–0393). The sharp diffraction peaks and absence of unknown peaks in the XRD spectrum of BiOBr demonstrate its high crystallinity and purity. Moreover, the diffraction peaks of Au-BiOBr are also similar to those of pure BiOBr, and only a weak peak, at 38.2°, found in the spectrum of the Au_0.9_-sample, belongs to the (111) facet of Au (S1 Fig). This finding indicates that the doped Au element does not affect the crystal phase of BiOBr frame, and Au might be highly dispersed in the host crystal. The peak of (001) facet at 10.9° is attributed to the periodic stacking structure among [Br-Bi-O-Bi-Br] layers along the c-axis, and the peak at 32.2° belongs to the (110) facet, which is perpendicular to (001) plane. For Au-doped BiOBr, the peak intensity of (001) facet increases while that of (110) facet decreases, indicating that the (110) facets were suppressed by Au doping.

**Fig 2:**
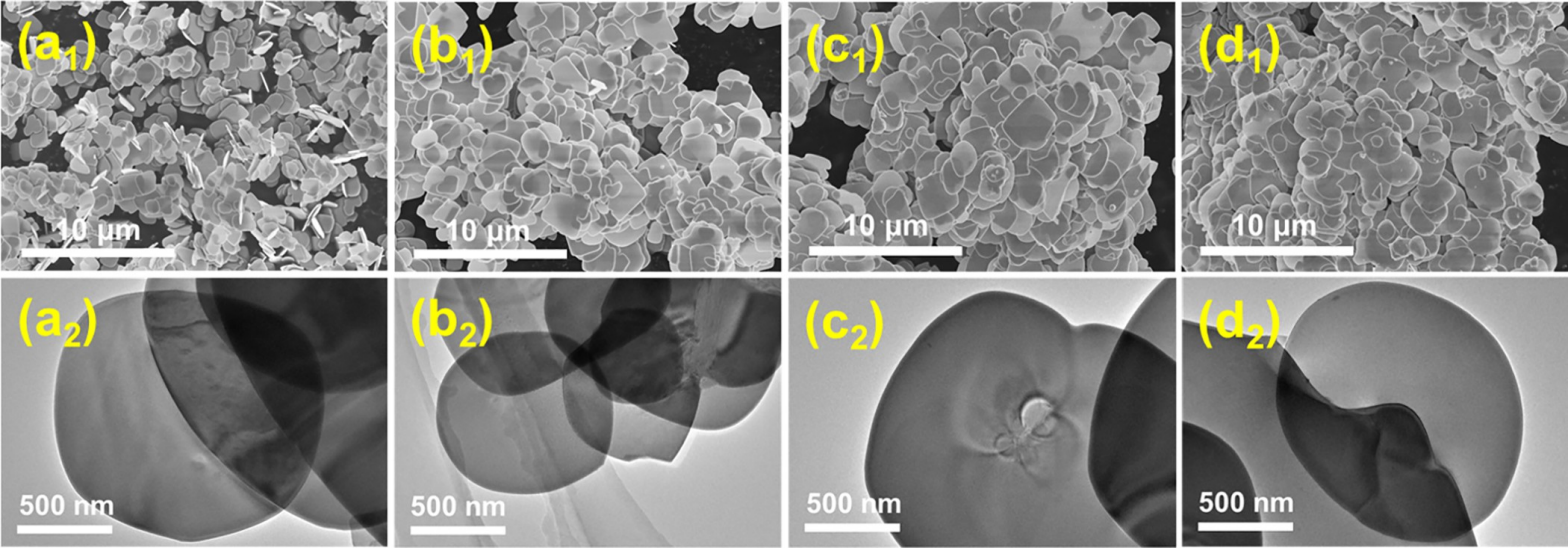
SEM and TEM images of (a_1_, a_2_) BiOBr, (b_1_, b_2_) Au0.3-BiOBr, (c_1_, c_2_) Au0.6-BiOBr and (d_1_, d_2_) Au0.9-BiOBr, respectively.

The morphology of the samples was observed by SEM and TEM (Fig 2). BiOBr and Au-doped BiOBr display a large-scale sheet-like structure. No impurities were observed in the images, indicating the high purity of the product, which is consistent with the conclusion from the XRD analysis. The products before and after modification by doping with Au had a mean size of 1 μm and a thickness of approximately 50 nm. This result implied that the doping of Au does not substantially alter the morphology of the products. Moreover, Au particles were not observed on the surface of the sheets in SEM and TEM images, which also indicated that Au might be doped into BiOBr crystal.

**Fig 3:**
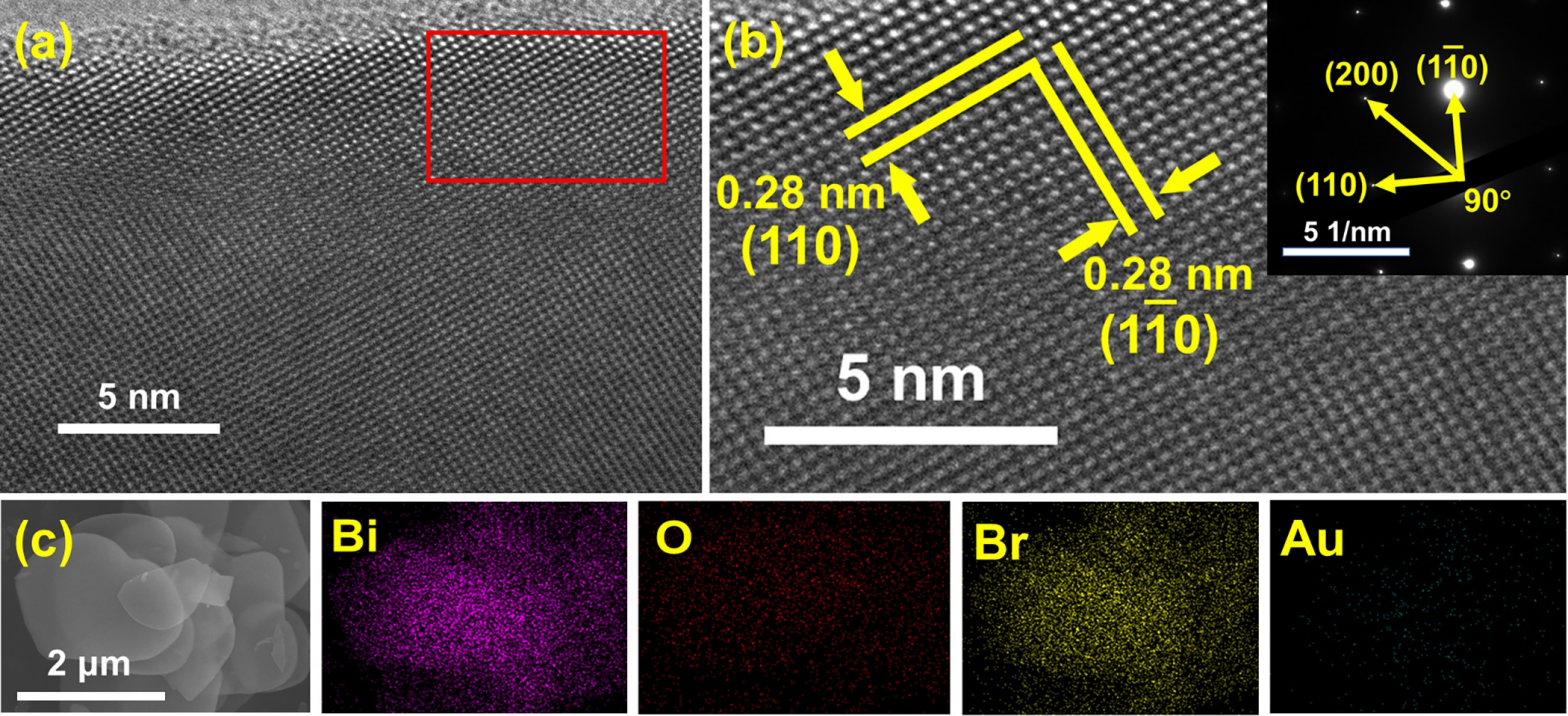
(a, b) HRTEM and (c) EDS mapping images of Au0.9-BiOBr. The insert in (b) is the corresponding SAED pattern.

The crystal structure of the as-prepared Au-doped BiOBr sheets was further characterized by high-resolution TEM (HRTEM) as shown in Fig 3. The image in Fig 3A shows the clear and continuous lattice fringes of the Au_0.9_-BiOBr sample. Also, as indicated in Fig 3B, the lattice fringes with an interplanar lattice spacing of 0.28 nm match well with the (110) atomic plane of BiOBr, whose diffraction peak in the XRD pattern occurred at 32.2°. In addition, the corresponding selected area electron diffraction (SAED) pattern (insert in Fig 3B) reveals the single-crystalline nature of BiOBr sheets and orthogonal to (110) and ($1\bar{1}0$) planes. Furthermore, the EDS mapping images (Fig 3C) revealed the highly homogeneous distribution of Bi, O, Br and Au elements in the as-prepared product, and the prsence of a very small amount of Au element in the crystal of BiOBr. Further analysis of the content of Au element in the product was by ICP-MS showed that the molar ratio of Au:Bi was approximately 0.01, 0.02 and 0.03 in Au_0.3_-BiOBr, Au_0.6_-BiOBr and Au_0.9_-BiOBr sheets, respectively. Thus, according to the above results, Au element was successfully introduced into the host crystal of BiOBr through the aforementioned one-pot hydrothermal procedure.

**Fig 4:**
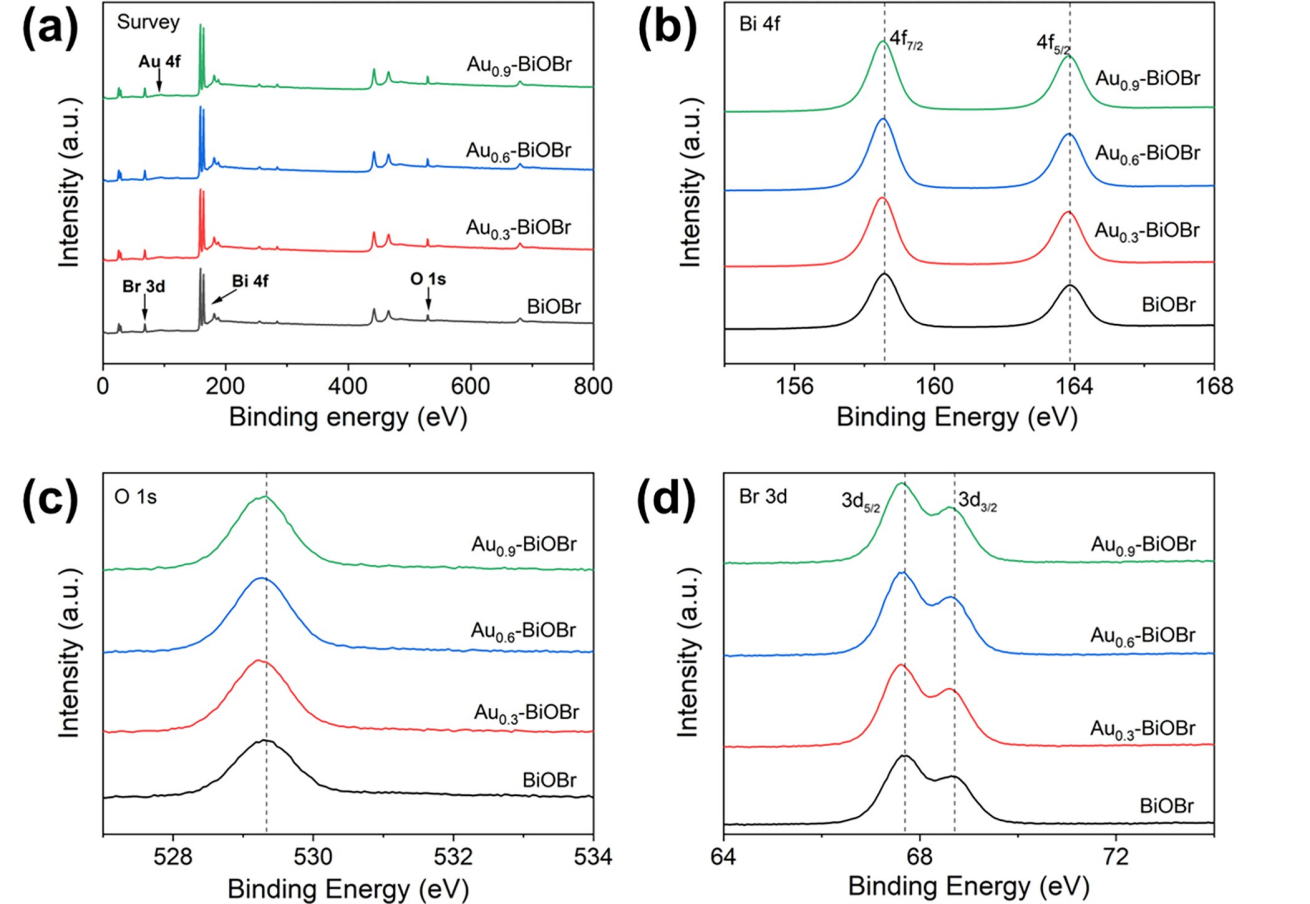
(a) XPS survey spectra, (b) Bi 4f, (c) O 1s, (d) Br 4f of BiOBr and Au-doped BiOBr.

**Fig 5:**
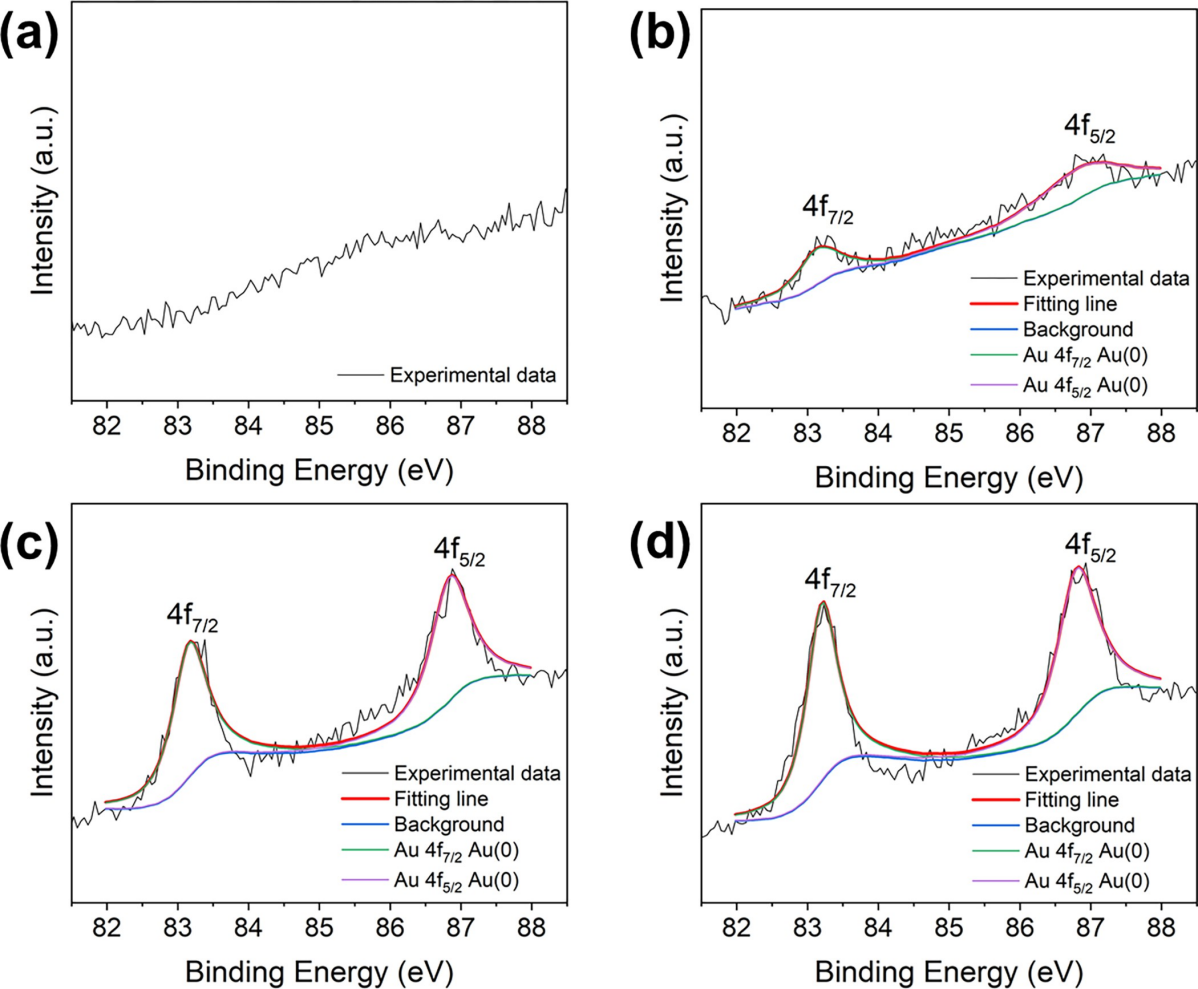
XPS spectra of Au 4f in (a) BiOBr, (b) Au0.3-BiOBr, (c) Au0.6-BiOBr and (d) Au0.9-BiOBr, respectively.

Characterization of the elemental composition and chemical states of the sample surface by XPS analysis, using the standard value of the C 1s peak (284.60 eV) to calibrate all samples, revealed that the spectra (Fig 4A) included the XPS peaks of Bi, O, Br and Au. The fine spectrum of Bi 4f (Fig 4B) contains two main peaks with binding energies of 158.5 and 163.8 eV, and with the splitting energy of 5.3 eV, which are consistent with the theoretical values of 4f_7/2_ and 4f_5/2_ of Bi^3+^ [9, 26, 27]. The XPS spectrum of O 1s (Fig 4C) shows that the strong peak position is at 529.3 eV, which corresponds to that of O_2_^-^ from the Bi-O bond [6, 28, 29]. Two peaks in the Br 3d XPS spectrum (Fig 4D) could be assigned to Br 3d_5/2_ (67.6 eV) and Br 3d_3/2_ (68.6 eV) of Br^-^ [9, 30]. The high-resolution Au 4f spectra are displayed in Fig 5. The two doublets at 83.2 and 86.8 eV are consistent with the binding energies of metallic Au 4f_7/2_ and Au 4f_5/2_, respectively, which are caused by metallic Au^0^ [31–33]. Therefore, all the aforementioned results demonstrate that the as-prepared product was an Au-doped BiOBr nanosheet with highly exposed (001) facet, and the amount of doped Au element in the host crystal was tunable.

### Band structures and electrochemical properties

**Fig 6:**
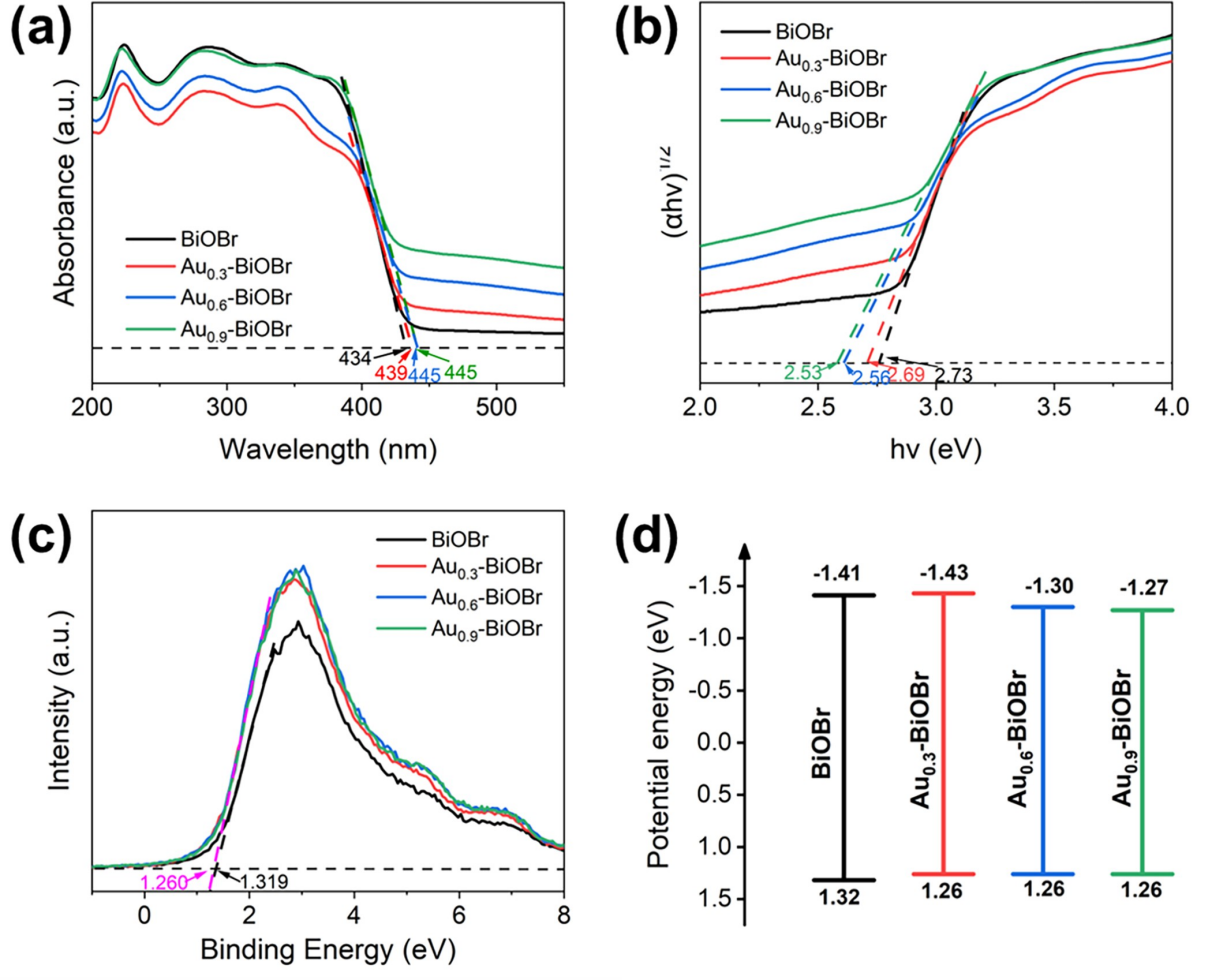
(a) UV-Vis DRS, (b) Tauc plots, (c) valence band and (d) band structure diagrams of BiOBr and Au-doped BiOBr, respectively.

The optical properties of the samples were characterized using UV-Vis diffuse reflectance spectroscopy (UV-Vis DRS), as shown in Fig 6A. The materials exhibit very similar absorption spectra. The absorption edges of BiOBr, Au_0.3_-BiOBr, Au_0.6_-BiOBr and Au_0.9_-BiOBr samples were 434, 439, 445 and 445 nm, respectively. The absorption edge of Au-doped BiOBr redshifted slightly compared to that of BiOBr, demonstrating their narrowed band gaps after Au-doping modification and higher absorption efficiency of visible light. Additionally, in the UV-Vis DRS spectra, no trailing were observed at about 550 nm, which indicates that the doped Au did not produce localized surface plasmon resonance (LSPR) effect. Specifically, the doped Au was highly dispersed in the BiOBr crystal, rather than forming heterojunctions on the surface of the BiOBr nanosheets.

The Tauc plot was used to determine the band gap energy of the semiconductor (Fig 6B), which was calculated by plotting the Tauc curve based on the relationship between light absorbance and band gap energy describe by Eq (1) [9]:

$$\alpha h\upsilon =A{\left(h\upsilon -E_{g}\right)}^{n/2}$$ (1)

where α, *hυ*, *A* and *E*_*g*_ are the absorption coefficient, incident photon energy, a constant and the energy of the band gap, respectively. Considering that BiOBr is a typical indirect band gap semiconductor, the *n* value is 4. The band gap energy of BiOBr, Au_0.3_-BiOBr, Au_0.6_-BiOBr and Au_0.9_-BiOBr were 2.73, 2.69, 2.56 and 2.53 eV, respectively. These findings indicate that after doping with Au the band gap of BiOBr became narrower, which is more suitable to harvest visible light.

The valance band (VB) top potential energy of the sample was measured using the XPS VB spectra (Fig 6C). The VB top of BiOBr was determined to be 1.32 eV. Coincidentally, samples doped with Au shared the same VB value of 1.26 eV. According to the semiconductor band gap energy and VB top potential energy, the CB bottom (*E*_CB_) of each sample was calculated using Eq (2) [26]:

$$E_{g}=E_{VB}-E_{CB}$$ (2)

where *E*_g_, *E*_VB_ and *E*_CB_ are the band gap energy, the VB top energy and the CB bottom energy, respectively. The band structure of each sample was determined, and the results are shown in Fig 6D. Clearly, after doping with Au, the band gap of the semiconductor was slightly narrowed. Also, the CB bottom energy would become less negative when more Au element was doped into the BiOBr crystal.

**Fig 7:**
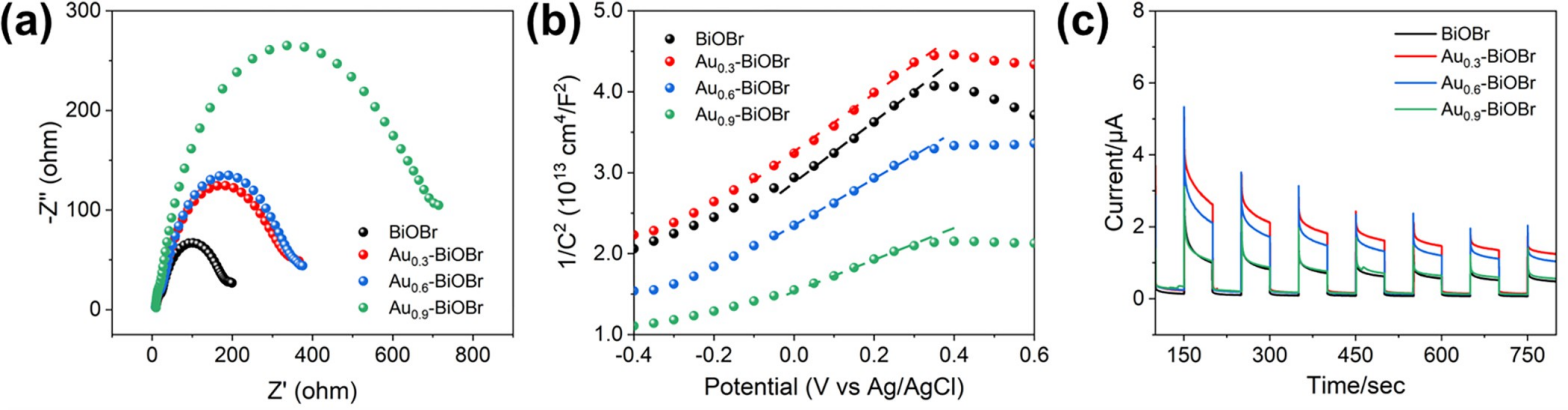
(a) EIS spectrum, (b) Mott-Schottky plots and (c) photocurrent response test plots of BiOBr and Au-doped BiOBr.

The electrochemical property is considered as another important factor that determine the performance of semiconductor photocatalysts. The EIS tests were performed to measure the efficiency of charge carrier separation and the transportation of the samples. As shown in Fig 7A, the curvature radius of the curves of the samples had the following order: Au_0.9_-BiOBr > Au_0.6_-BiOBr > Au_0.3_-BiOBr > BiOBr. These results showed that the curvature radius increases with the increase of the Au doping concentration. Specifically, modification by doping with Au enhanced the resistance of BiOBr.

Furthermore, Mott-Schottky plots (Fig 7B) were used to determine the density of charge carriers of the samples using Eq (3) [6]:

$$N_{d}=\left(\frac{2}{e_{0}\varepsilon \varepsilon_{0}}\right){\left[\frac{d(C^{2})}{dV}\right]}^{-1}$$ (3)

where *e*_0_, *ε* and *ε*_0_ are the electron charge, the dielectric constant of the sample and the permittivity of the vacuum, respectively; [d(*C*^*2*^)/d*V*]^-1^ is the slope of the Mott-Schottky plots. Clearly, the linear part of the Mott-Schottky curve had a positive slope, revealing that all the as-prepared products were *n*-type semiconductors. The slopes of these samples were in the following order: Au_0.3_-BiOBr > BiOBr > Au_0.6_-BiOBr > Au_0.9_-BiOBr. Given that the carrier concentration is inversely proportional to the slope of the linear part of the Mott-Schottky curve, the carrier concentration of Au_0.3_-BiOBr slightly decreased by 7% compared with that of BiOBr. When more Au element was introduced, the carrier concentration of the semiconductor was substantially increased.

In addition, transient photocurrent response measurements (Fig 7C) were performed to investigate the charge injection properties of the as-prepared photocatalysts. Considering that BiOBr only weakly absorbs visible light, UV light was used in this experiment to compare the charge injection characteristics of Au-doped BiOBr and BiOBr. The order of the photocurrent response intensity of these photocatalysts was as follows: Au_0.3_-BiOBr > Au_0.6_-BiOBr > Au_0.9_-BiOBr > BiOBr. This order reveals that Au0.3-BiOBr nanosheets showed the highest photocurrent, which was 2.2 times higher than that of BiOBr. Therefore, according to the results of carrier concentration, the charge separation efficiency of Au_0.3_-BiOBr was 2.3 times higher than that of BiOBr, demonstrating that modification by doping with Au could substantially improve the charge separation efficiency of the catalyst.

### Photocatalytic degradation of TH

**Fig 8:**
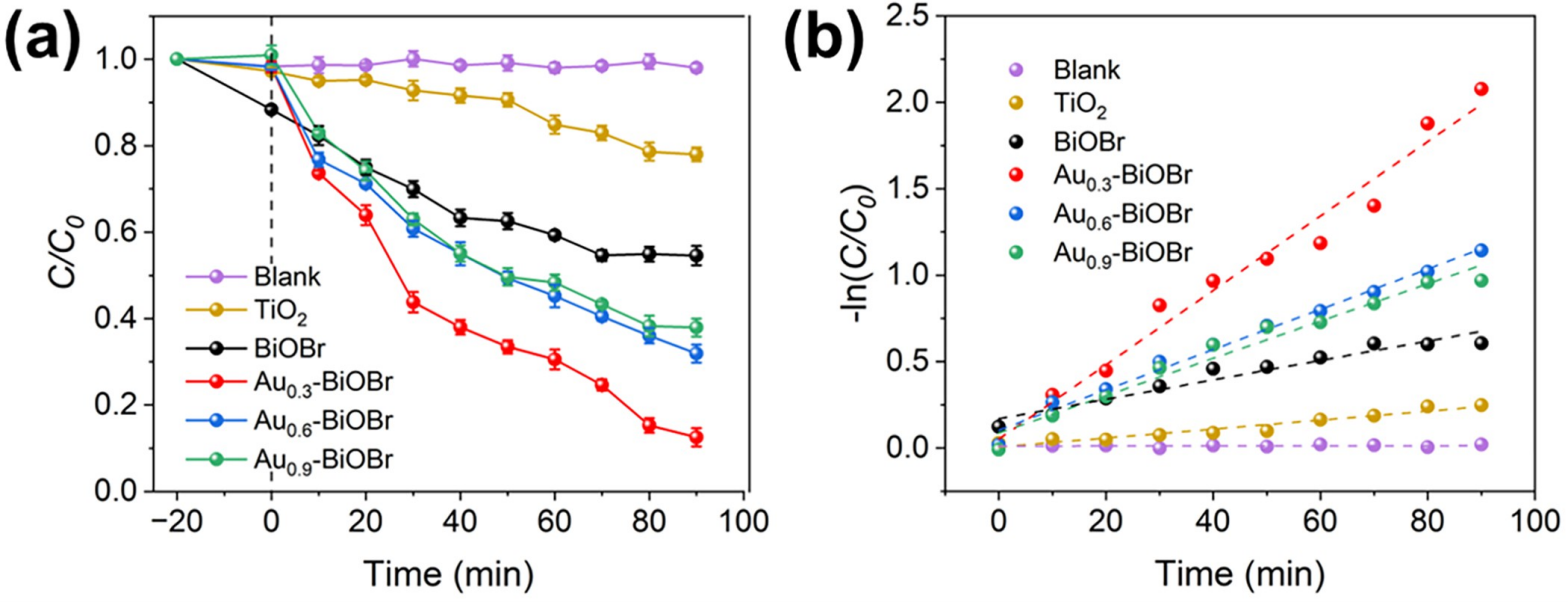
(a) Photocatalytic degradation curves and (b) corresponding kinetic curves of the BiOBr and Au-doped BiOBr, respectively.

The degradation efficiency of TH was investigated to evaluate the photocatalytic performance of various catalysts under visible light irradiation. The degradation trend of TH in the presence of different catalysts during 90 min of irradiation is shown in Fig 8A. To achieve the adsorption/resolution equilibrium of the photocatalyst, the reaction system was stirred in the dark for 20 minutes. In the absence of photocatalyst, the decomposition of TH was negligible, indicating that TH is very stable under visible light irradiation. The degradation efficiency of TH by TiO_2_ and BiOBr was only 21.5 and 44.8%, respectively, indicating that the photocatalytic degradation of TH by TiO_2_ and BiOBr under visible light irradiation were limited. However, after modification by doping with Au, the TH degradation efficiency for Au_0.3_-BiOBr, Au_0.6_-BiOBr and Au_0.9_-BiOBr was 88, 67.5 and 62.4%, respectively. It is evident that Au_0.3_-BiOBr exhibited the highest rate of degradation of TH under visible light irradiation and thus had the best performance among these photocatalysts. A table is presented to compare organics removal over different systems and makes a comparison with this work (S1 Table) [9, 34–36]. Combined with the electrochemical characteristics of these samples, these results revealed that modification by doping with Au mainly reduces the band gap of the semiconductor and increases the carrier concentration, but it also increases the impedance, which is not conducive to carrier migration. As a result, the Au_0.9_-BiOBr nanosheets with the narrowest band gap did not have the highest photocatalytic efficiency due to its low charge separation efficiency. However, in the case of Au_0.3_-BiOBr nanosheets, the highest photocatalytic degradation efficiency was achieved due to the substantially enhanced visible light absorption efficiency and charge separation efficiency of Au_0.3_-BiOBr compared to those of BiOBr.

The following pseudo first-order kinetics Eq (Eq 4) was used to quantitatively describe the photocatalytic efficiency of these samples [9]:

$$-\ln\left(C_{t}/C_{0}\right)=kt$$ (4)

where *C*_t_, *C*_0_, *k* and *t* are the concentration at the given time *t*, initial concentration, the kinetic constant and time, respectively. The corresponding first order dynamics diagram is shown in Fig 8B. The *k* values for TiO_2_, BiOBr, Au_0.3_-BiOBr, Au_0.6_-BiOBr and Au_0.9_-BiOBr were 0.0025, 0.0055, 0.0216, 0.0116 and 0.0109 min^-1^, respectively. The photocatalytic efficiency of Au_0.3_-BiOBr was 3.9 times higher than that of BiOBr. In addition, the specific values of the BET surface area for BiOBr, Au_0.3_-BiOBr, Au_0.6_-BiOBr and Au_0.9_-BiOBr were 2.15, 4.31, 2.65 and 2.65 m^2^ g^-1^, respectively (S2 Fig). Additionally, the surface-area-normalized kinetic constant (*k*/*S*_BET_) was calculated to eliminate differences in the exposure of active sites. The *k*/*S*_BET_ values of BiOBr, Au_0.3_-BiOBr, Au_0.6_-BiOBr and Au_0.9_-BiOBr were 2.56, 5.01, 4.38 and 4.11 mg m^-2^ min^-1^, respectively. Thus, the intrinsic photocatalytic efficiency of Au_0.3_-BiOBr was approximately twice higher than that of BiOBr, indicating that modification by doping with Au could enhance the intrinsic photocatalytic activity of BiOBr by improving the charge separation efficiency under visible light irradiation. Furthermore, S3 Fig shows the stability of Au_0.3_-BiOBr nanosheets in the photocatalytic degradation of TH under visible light irradiation. After 4 circulations of TH degradation, the photocatalytic activity of Au_0.3_-BiOBr nanosheets retained about 95.2%, confirming the high stability of the photocatalytic activity. Moreover, to further demonstrate the stability of the catalyst itself, the XRD and SEM were used to analyze the phase and morphology of the Au_0.3_-BiOBr nanosheets after reaction (S4 Fig). The XRD pattern of sample after reaction was almost unchanged compared to the unused Au_0.3_-BiOBr nanosheets, which indicates the high stability of crystal structure.

### Mechanism of TH photocatalytic degradation

**Fig 9:**
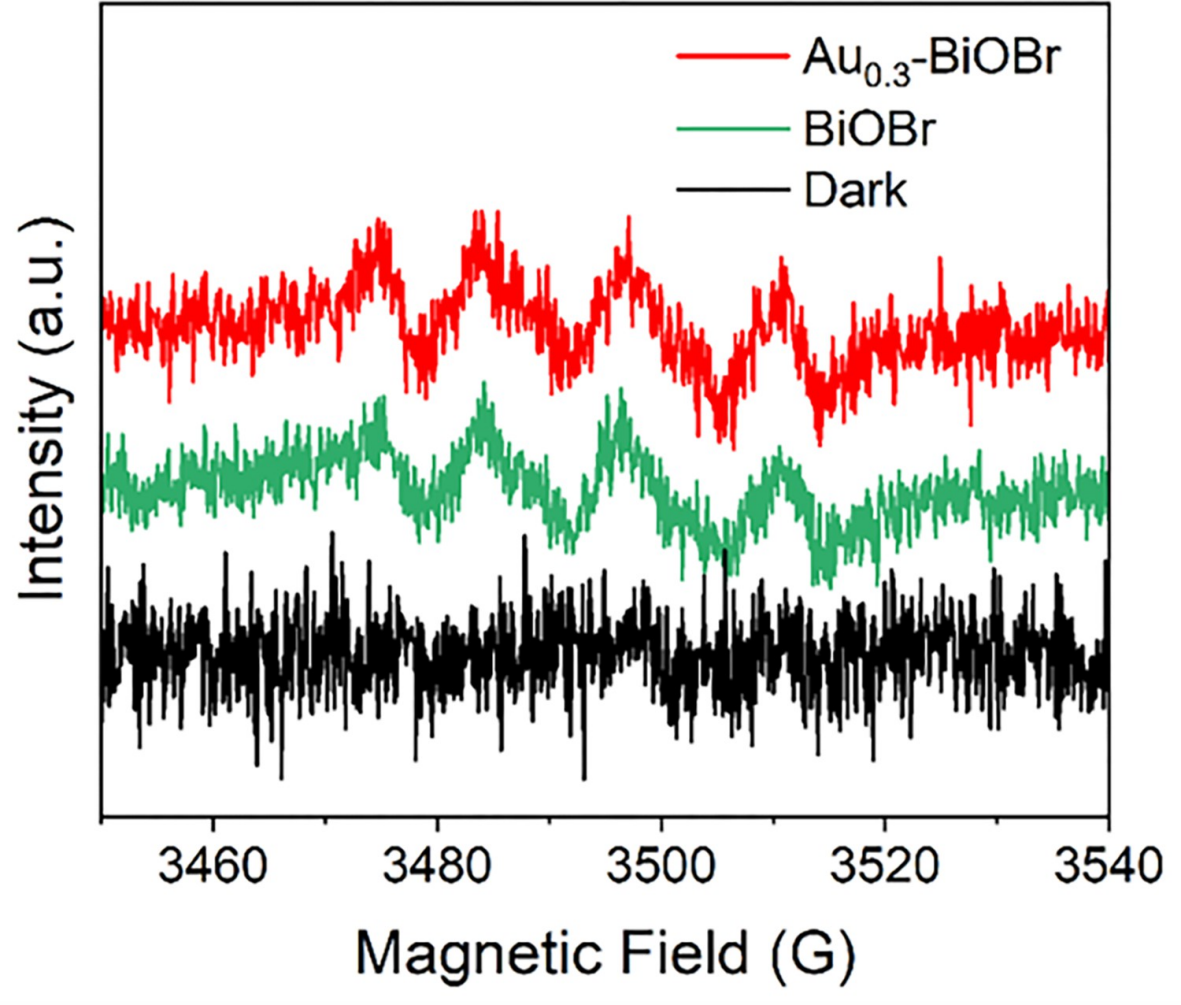
EPR spectrum of ·O2-.

Due to the strong oxidation capacity of reactive oxygen species (ROS) such as ·O_2_^-^, they usually play an important role in the photocatalytic degradation process. Therefore, to verify the presence of ·O_2_^-^, nitroblue tetrazolium (NBT) was chosen to trap the ·O_2_^-^ generated in the visible light irradiation over the Au_0.3_-BiOBr nanosheets, and the result was shown in S5 Fig. The maximum absorbance of NBT declined prominently, indicating that abundant ·O_2_^-^ has been generated rapidly under visible light [29]. Furthermore, electron paramagnetic resonance (EPR) spectroscopy analysis was performed to investigate the active substances produced during the photocatalytic degradation of TH. The corresponding EPR spectra, shown in Fig 9, reveal that, under visible light irradiation, ·O_2_^-^ was generated in samples with Au_0.3_-BiOBr and BiOBr. The signal of ·O_2_^-^ generated with Au_0.3_-BiOBr was slightly stronger than that with BiOBr, indicating that more ·O_2_^-^ was generated with Au_0.3_-BiOBr during the photocatalytic degradation process.

**Fig 10:**
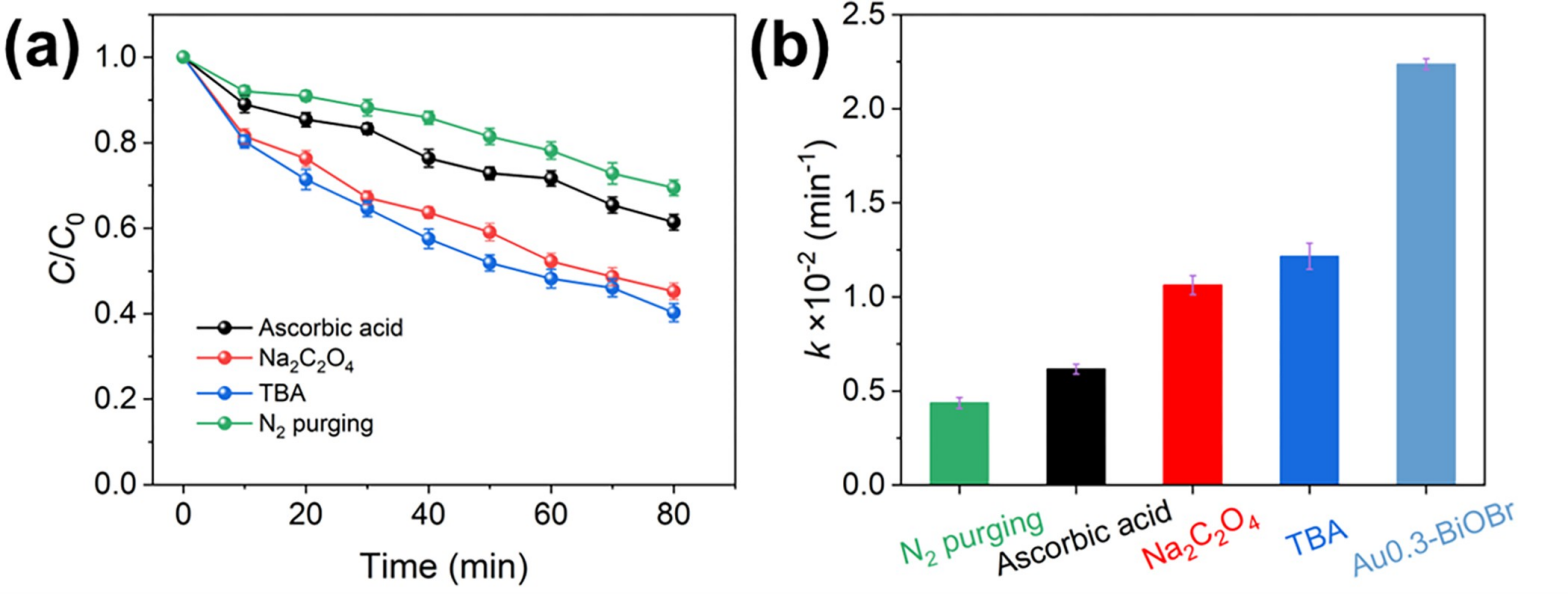
(a) photocatalytic degradation curves of TH with added scavengers and (b) the corresponding kinetic constants.

The difference of the effects of the main active substances on the photocatalytic degradation of TH was investigated through a series of free radical quenching experiments using the scavengers N_2_, ascorbic acid, Na_2_C_2_O_4_ and tert-butyl alcohol (TBA), to remove dissolved O_2_, ·O_2_^-^, h^+^ and ·OH, respectively. As is shown in Fig 10A, these four scavengers inhibited the degradation of TH to different degrees. In particular, N_2_ purging and ascorbic acid had a strong effect on the degradation of TH. The kinetic constants of TH degradation with the scavengers N_2_, ascorbic acid, Na_2_C_2_O_4_ and TBA were 0.005, 0.0056, 0.009 and 0.0101, respectively (Fig 10B). Compared with the kinetic constants of TH degradation without scavengers, these scavengers reduced the kinetic constants by 77, 74, 58 and 53%, respectively, confirming that ·O_2_^-^ was the main active species in the TH degradation process. However, the contribution of h^+^ and ·OH during the TH degradation process should not be neglected. The generated ·O_2_^-^ could be transformed into ·OH *via* the following reactions [4, 6, 9]:

$$O_{2}+e^{‐}\to \cdot O_{2^{‐}}$$ (5)

$$\cdot O_{2^{‐}}+H_{2}O\to \cdot \mathrm{OOH}+{\mathrm{OH}}^{‐}$$ (6)

$$\cdot \mathrm{OOH}+H_{2}O+2e^{‐}\to \cdot \mathrm{OH}+2{\mathrm{OH}}^{‐}$$ (7)

**Fig 11:**
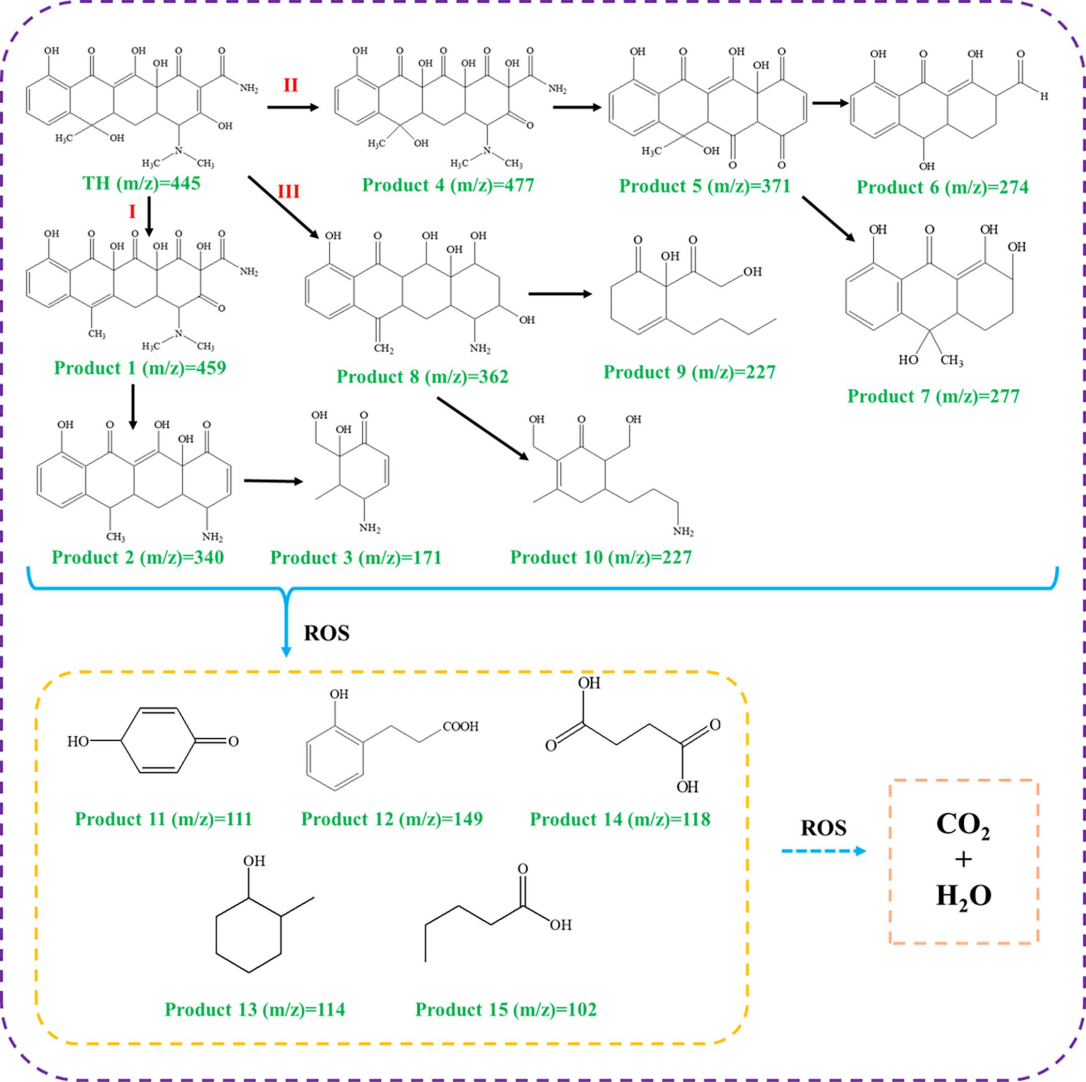
Proposed degradation pathways of TH by the Au0.3-BiOBr under the visible light.

The analysis of the intermediates formed in the degradation process by HPLC-MS revealed the pathways for the photocatalytic degradation of TH. According to the results of the analysis of intermediates, the TH degradation process could be divided into four stages, namely hydroxylation, demethylation, ring opening and mineralization. Some functional groups in the TH molecule, such as double bond, phenol group and amine group, have a high electron density and are susceptible to ROS during the degradation process, resulting in a series of intermediates [37, 38]. Therefore, as illustrated in Fig 11, three pathways for TH degradation were identified. The initial *m/z* value of the original TH was 445. Through the hydroxylation reaction, intermediates with *m/z* of 459 and 477 were generated. Then, due to the continuous attack by ROS, demethylation occurs and breaks the amino, methyl and hydroxyl groups, which result in the generation of intermediates with *m/z* of 340, 362 and 371. Through aromatic ring-opening reactions, the intermediates with *m/z* of 171, 227, 274 and 277 were generated followed by intermediate with *m/z* of 340, 362 and 371. As a result of the occurrence of further aromatic ring-opening reactions and the absence of certain functional groups, some intermediates were further degraded into small molecule compounds with *m/z* of 111, 149, 114, 118 and 102. Ultimately, these small compounds were continuously decomposed into even smaller compounds until completely mineralized to CO_2_ and H_2_O.

Based on the above results, the elucidated mechanism of photocatalytic TH degradation by Au-doped BiOBr nanosheets under visible light irradiation can be summarized as follows. First, Au-doped BiOBr nanosheets absorb visible light and become excited to generate electrons and holes. Then, the h^+^-e^-^ pairs are efficiently separated in the crystal of Au_0.3_-BiOBr. After that, free radicals including ·O_2_^-^ and ·OH are generated by the injection of e^-^ into O_2_, which are responsible for the degradation of TH. In this way, TH is excited by these free radicals and degraded into intermediates, and eventually mineralized to CO_2_ and H_2_O.

## Conclusions

In this study, Au-doped BiOBr nanosheets were successfully synthesized through a simple one-pot hydrothermal method, and the Au atoms were found to be highly dispersed in the BiOBr host crystal. Au_0.3_-BiOBr nanosheets had a narrower band gap and a higher charge separation efficiency than those of BiOBr. Specifically, the charge separation efficiency of Au_0.3_-BiOBr was 2.3 times higher than that of BiOBr. Therefore, Au_0.3_-BiOBr nanosheets exhibited the highest efficiency in the degradation of TH under visible light irradiation, which was twice higher than that of BiOBr. During the photocatalytic process, ·O_2_^-^ was the main active species while other ROS also contributed to the degradation of TH. Furthermore, the degradation pathways of TH were elucidated, and shown to involve hydroxylation, demethylation, ring opening and mineralization. Therefore, modification by doping with Au is proposed as a promising strategy for the modification of BiOBr to improve the charge separation efficiency and enhance the photocatalytic degradation efficiency for organic pollutants under visible light irradiation.

## Supporting information

S1 Fig XRD patterns of Au0.9-BiOBr. (TIF)

S2 Fig Nitrogen adsorption/desorption isotherms of the BiOBr and Au-doped BiOBr, respectively. (TIF)

S3 Fig Recycling properties of the photocatalytic degradation of TH over the Au_0.3_-BiOBr nanosheets. (TIF)

S4 Fig (a) XRD spectra and (b) SEM image after several cycles of photocatalytic degradation of TH over the Au_0.3_-BiOBr nanosheets.
(TIF)

S5 Fig Absorbance of NBT curve with time over Au_0.3_-BiOBr nanosheets. (TIF)

S1 Table Comparison of degradation capacities of some catalysts as described in literature. (DOCX)

S1 Graphical abstract (TIF)
