# Supplementary figures and images for: Enhanced photocatalytic degradation of tetracycline hydrochloride over Au-doped BiOBr nanosheets under visible light irradiation
Source: PLoS One. 2022 Aug 26;17(8):e0273169. doi: 10.1371/journal.pone.0273169 (PMC9417003; doi:10.1371/journal.pone.0273169)

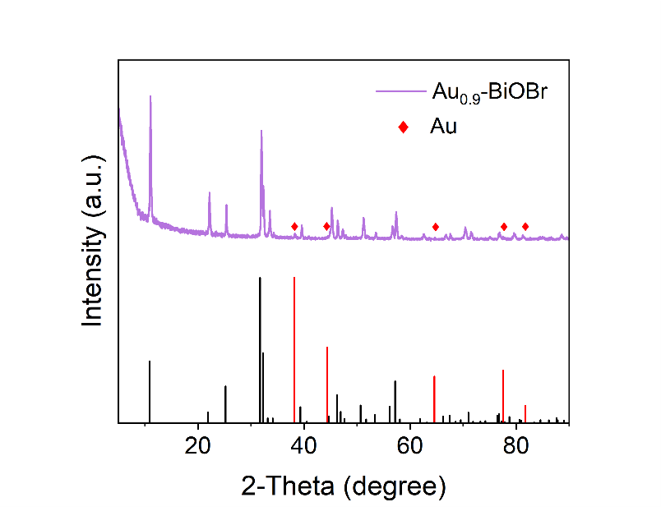

Supplement: S1 Fig — (TIF) [file pone.0273169.s001.tif]

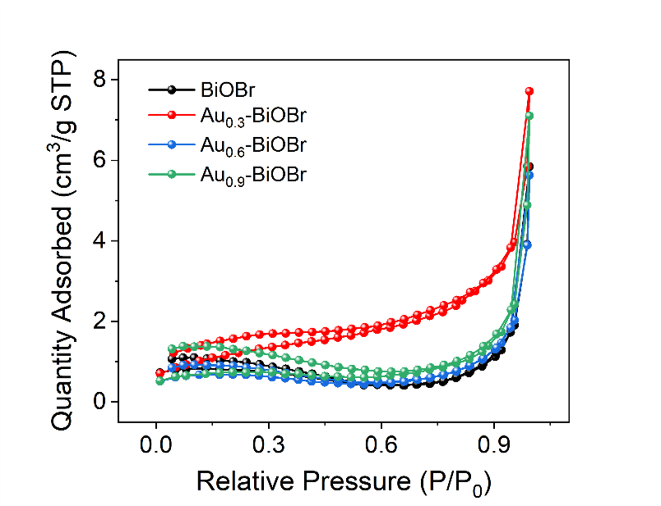

Supplement: S2 Fig — (TIF) [file pone.0273169.s002.tif]

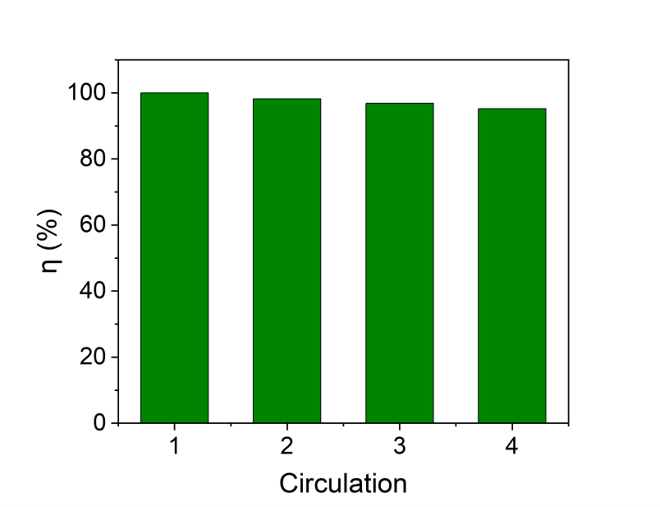

Supplement: S3 Fig — (TIF) [file pone.0273169.s003.tif]

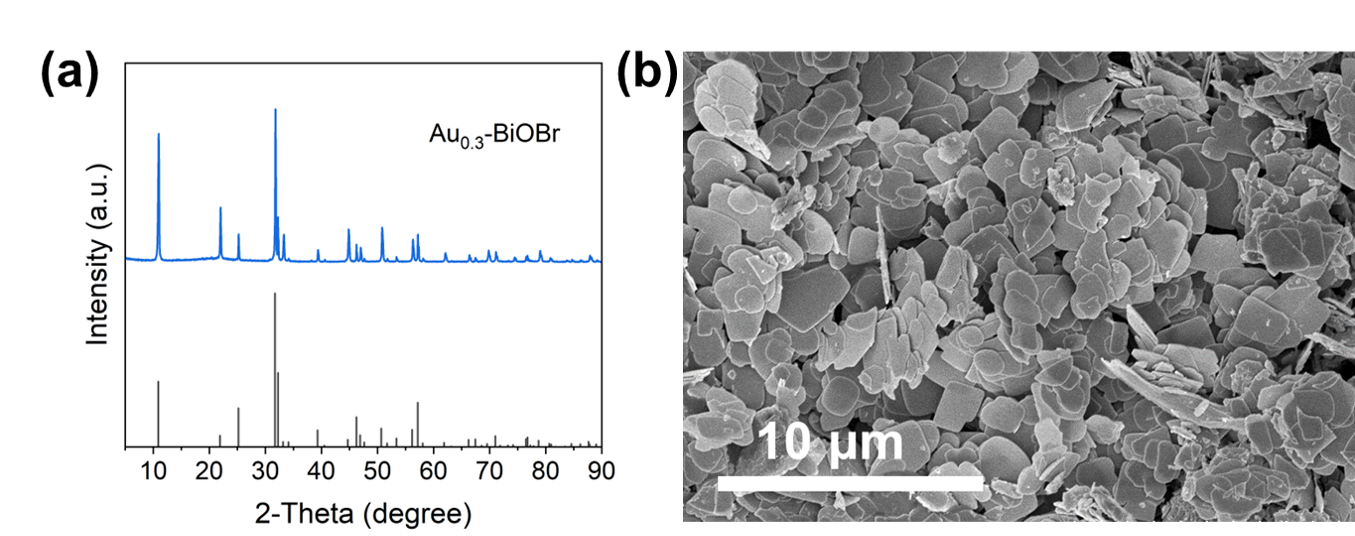

Supplement: S4 Fig — (a) XRD spectra and (b) SEM image after several cycles of photocatalytic degradation of TH over the Au0.3-BiOBr nanosheets. (TIF) [file pone.0273169.s004.tif]

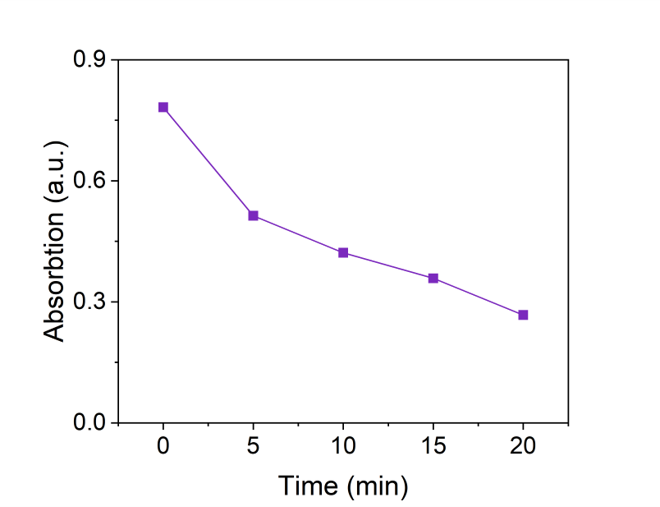

Supplement: S5 Fig — (TIF) [file pone.0273169.s005.tif]

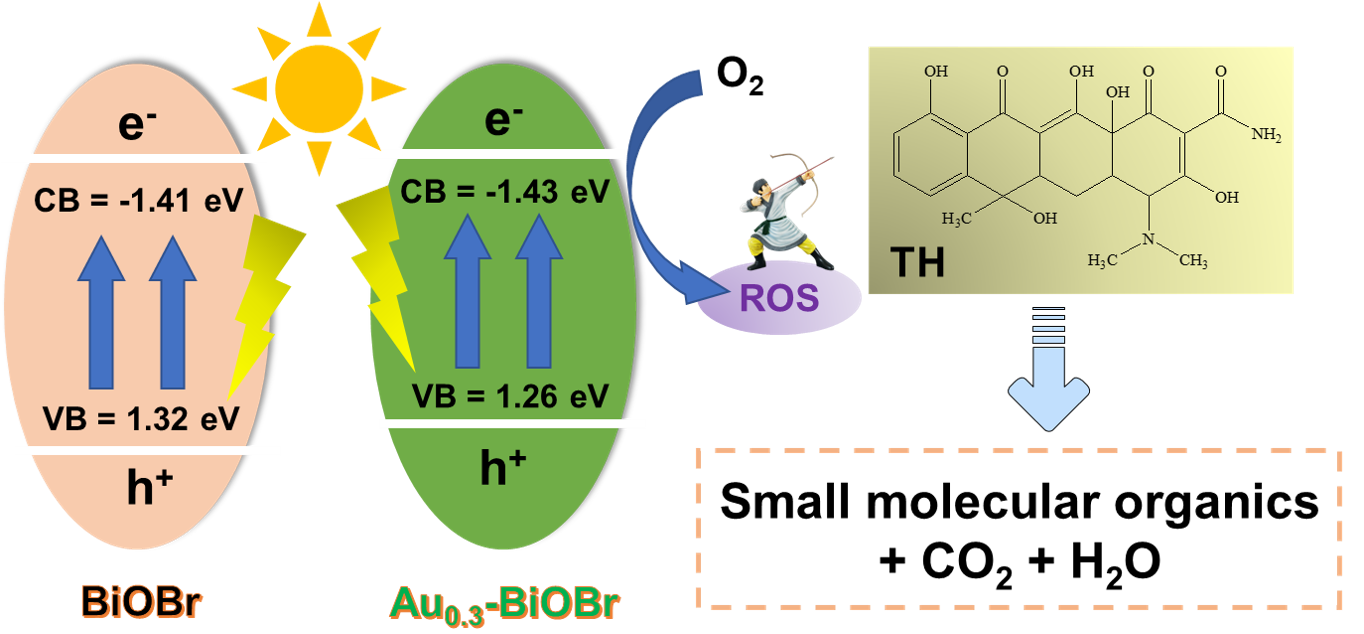

Supplement: S1 Graphical abstract — (TIF) [file pone.0273169.s007.tif]
