# Supplementary material for: Enhanced photocatalytic degradation of tetracycline hydrochloride over Au-doped BiOBr nanosheets under visible light irradiation
Source: PLoS One. 2022 Aug 26;17(8):e0273169. doi: 10.1371/journal.pone.0273169 (PMC9417003; doi:10.1371/journal.pone.0273169)
Supplement: S1 Table — (DOCX) [file pone.0273169.s006.docx]

| Materials | Concentration of catalyst | | Organics | Concentration of organics (mg/L) | Degradation time (h) | Degradation rate (%) | References |
| --- | --- | --- | --- | --- | --- | --- | --- |
| Au_0.3_-BiOBr | | 0.2 g/L | TH | 50 | 1.5 | 88 | This work |
| S_0.2_-BiOBr | | 0.25 g/L | BPA | 10 | 3 | 92 | [3] |
| BiOCl@CeO_2_ | | 0.5 g/L | TC | 10 | 2 | 92 | [34] |
| ZnO_2_ | | 0.1 g/L | TC | 50 | 4 | 83.7 | [35] |
| GNG8+PMS | | 0.05 g/L + 0.2 mM | RhB | 20 | 1 | 100 | [36] |
